# Challenges and Therapeutic Opportunities of Autophagy in Cancer Therapy

**DOI:** 10.3390/cancers12113461

**Published:** 2020-11-20

**Authors:** Valdenizia R. Silva, Sara P. Neves, Luciano de S. Santos, Rosane B. Dias, Daniel P. Bezerra

**Affiliations:** Gonçalo Moniz Institute, Oswaldo Cruz Foundation (IGM-FIOCRUZ/BA), Salvador 40296-710, Brazil; valdeniziar@gmail.com (V.R.S.); saraparenteneves@gmail.com (S.P.N.); luciano.biomed@gmail.com (L.d.S.S.); rosanebd@gmail.com (R.B.D.)

**Keywords:** autophagy, cancer, cell death, drug resistance

## Abstract

**Simple Summary:**

Autophagy is a physiological process characterized by the degradation of the cell components through lysosomes due to stimuli/stress. In this study, we review the challenges and therapeutic opportunities that autophagy presents in the treatment of cancer. We discussed the results of several studies that evaluated autophagy as a therapeutic strategy in cancer, both through the modulation of therapeutic resistance and the death of cancer cells. Moreover, we discussed the role of autophagy in the biology of cancer stem cells and the inhibition of this process as a strategy to overcome resistance and progression of cancer stem cells.

**Abstract:**

Autophagy is a physiological cellular process that is crucial for development and can occurs in response to nutrient deprivation or metabolic disorders. Interestingly, autophagy plays a dual role in cancer cells—while in some situations, it has a cytoprotective effect that causes chemotherapy resistance, in others, it has a cytotoxic effect in which some compounds induce autophagy-mediated cell death. In this review, we summarize strategies aimed at autophagy for the treatment of cancer, including studies of drugs that can modulate autophagy-mediated resistance, and/or drugs that cause autophagy-mediated cancer cell death. In addition, the role of autophagy in the biology of cancer stem cells has also been discussed.

## 1. Introduction

Autophagy is a catabolic process, strongly regulated, necessary for the maintenance of biological functions. This process occurs when autophagic vacuoles fuse with lysosomes to form the autophagolysosome, which digests its own cellular components through lysosomal enzymes, degrading them for subsequent generation of cellular energy in vital processes and also eliminating damaged and poorly organized proteins [1,2]. The autophagic mechanism is correlated with several cellular signaling pathways and is activated by autophagy-related genes (ATG), triggered in physiological conditions as a response to different stimuli/stress, such as nutrient deprivation and oxidative stress. Therefore, autophagy is important for genomic stability and protection against various diseases [3].

Autophagy plays a dual role in cancer—while in some situations it has a cytoprotective effect, in others, it has a cytotoxic effect. Therefore, the modulation of the autophagic flux can be a therapeutic strategy for cancer. However, autophagic outcomes in different types of cancer depend on the context and are not yet fully defined, which makes its clinical application difficult [4]. In addition, tumors use various chemotherapy resistance strategies, such as activating alternative pathways, increasing drug efflux and mutations in the drug target [5].

Cytoprotective autophagy is also one of the mechanisms of resistance to chemotherapy, since, in response to the hostile environment, different pathways can regulate autophagy as a cell survival mechanism. In addition, autophagy can regulate the properties of cancer stem cells, contributing to the maintenance of the characteristics of stem cells [6,7]. Therefore, to overcome chemoresistance, autophagy modulators and their related pathways can be used to sensitize cancer cells to currently available treatments.

On the other hand, autophagy may exhibit anticancer properties due to a hyperactivation, causing cell death mediated by autophagy, which can occur with the absence of the pro-apoptotic proteins B-cell lymphoma 2 (BCL-2)-associated X (BAX) and BCL-2 homologous antagonist/killer (BAK) [8,9,10]. Genes related to autophagy are also needed in the cell death by autosis, which is mediated by the Na^+^, K^+^-ATPase pump [11]. In this context, autophagy machinery are also needed for chemotherapy action. Thus, understanding the role of autophagy in each cell context and underlying signaling pathways is crucial for regulating the impact of autophagy on cell death or survival.

In this review, we summarize strategies aimed at autophagy for cancer treatment, including studies of drugs related to modulating autophagy-mediated resistance and/or drugs that can cause autophagy-mediated cancer cell death. Furthermore, the role of autophagy in the biology of cancer stem cells has also been discussed.

## 2. Cellular and Molecular Aspects of Autophagy

Autophagy is an evolutionarily conserved physiological cellular process in response to different stimuli/stress, including nutrient deprivation or metabolic disorders. It is a mechanism involved in the degradation and elimination of intracellular aggregates and damaged organelles, allowing the basal turnover of cellular components, and providing energy and macromolecular precursors, in addition to eliminating intracellular pathogens [12,13,14].

The autophagic catabolic process was first defined by Christian De Duve more than 50 years ago during a study that used liver perfused with glucagon, with the name meaning “self-feeding” (from the Greek words “auto” meaning self and “phagy” meaning feeding) [15].

Autophagy overcomes the pathophysiological process to maintain homeostasis, cellular biological function, metabolism, and cell survival, being the main process of degradation and intracellular recycling [16,17]. Dysfunctional autophagy is associated with axonal degeneration in several neurodegenerative diseases [18], inflammation and inflammatory diseases [19], cardiovascular diseases [20], immune diseases [21], and cancer [22].

In particular, the first link between autophagy and a human disease was made by Levine et. al., who first sequenced the *BECN1* gene (which encodes the beclin-1/ATG6 protein), followed by the discovery that a *BECN1* allele is often deleted in some types cancer and that beclin-1 induces autophagy and inhibits tumor formation in human breast cancer cell line MCF-7 [23,24,25]. Mutation in beclin-1 also reduces its capability to suppress tumorigenesis [26]. In this context, Marino et. al. [27] demonstrated that atg4c^−/−^ (in all tissues) mice presented increased fibrosarcoma induced by chemicals. Furthermore, atg5f^/F^:nestin-Cre (in neurons) mice presented progressive neurodegeneration associated with ubiquitinated protein aggregates and inclusion bodies [28], and atg5f^/F^:MLC2v-Cre (in cardiomyocytes) mice, although presenting normal hearts under basal conditions, showed increased pressure load-induced ventricular dilatation and heart failure [29]. Beclin-1^−/−^ (in all tissues) mice die early in embryogenesis and beclin-1^−/+^ increased frequency of spontaneous malignancies, especially lymphomas, decreased pressure overload-induced heart failure, and decreased cardiac injury during ischemia/reperfusion [30,31,32,33]. Atg7^−/−^ (in all tissues) mice died 24 h after birth, probably due to depletion of nutrients and energy [34], while atg7f^/F^:nestin-Cre (in neurons) mice developed progressive neurodegeneration associated with ubiquitinated protein aggregates and inclusion bodies, and increased frequency of neuron death [35]; and atg7f^/F^:Mx1-Cre (in liver) mice presented ubiquitinated protein aggregates, deformed mitochondria, and aberrant membranous structures in hepatocytes along with reduced removal of peroxisomes after chemical treatment [34,36].

Endogenous and exogenous stimuli/stress induce autophagy for degradation or as a repair mechanism [37]. This stimuli/stress include glucose or amino acid deprivation, amino acid metabolite of ammonium, iron depletion, absence of growth factors, hypoxia, endoplasmic reticulum (ER) stress, oxidative stress, and infection by pathogens [38,39,40,41,42,43,44,45].

### 2.1. Autophagy Types

The autophagic process is categorized into three types on the basis of molecular machinery, morphological characteristics, and mechanisms by which intracellular components are delivered to degradation: chaperone-mediated autophagy, microautophagy, and macroautophagy [46,47,48,49].

**Figure 1:**
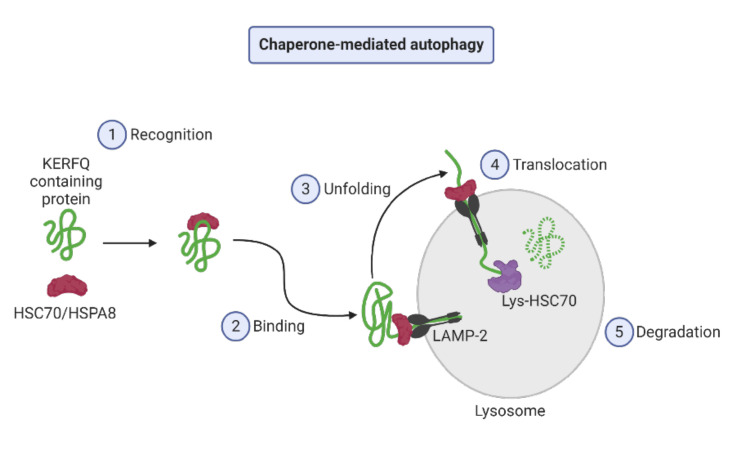
Steps of chaperone-mediated autophagy.

Chaperone-mediated autophagy (CMA) (Figure 1) was the first lysosomal process to be discovered by which intracellular components are selectively degraded [50]. Soluble proteins are selectively degraded by lysosomes in the CMA subtype. The C-terminal pentapeptide KFERQ motif is recognized by the cytoplasmic chaperone heat shock cognate 71 kDa protein (HSC70, also known as HSPA8) that interacts with the lysosome-associated membrane protein type 2a (LAMP2A) and leads to protein targets for degradation [51,52]. The degradation of specific cellular proteins through a dedicated translocation complex in the CMA modulates glucose and lipid metabolism, DNA repair, cellular reprogramming, and the cellular response to stress [53].

**Figure 2:**
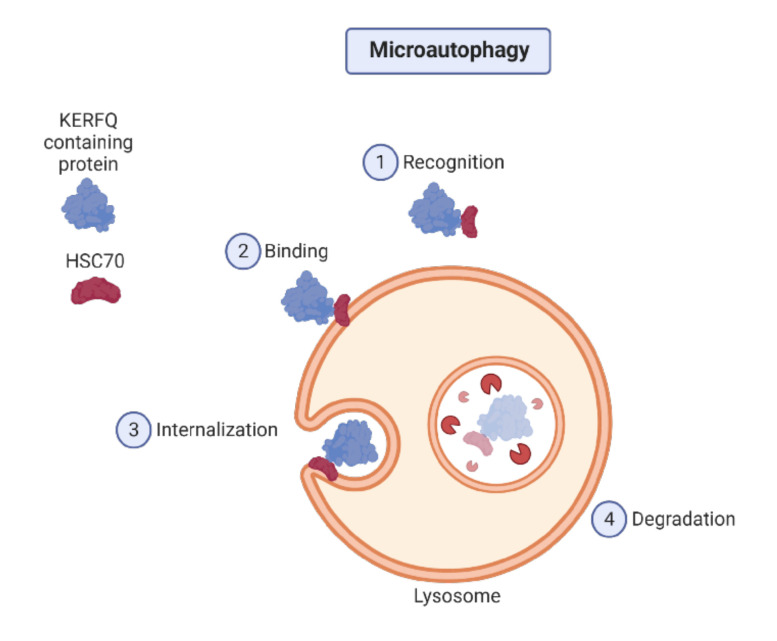
Steps of microautophagy.

Microautophagy involves the invagination of the lysosomal membrane, which involves the cytosolic components and seems skewed towards aberrant or inactive proteasomes [54]. Maintaining the size of organelles, membrane homeostasis, and cell survival under nitrogen restriction are the main functions of microautophagy [55]. The steps of microautophagy are shown in Figure 2.

**Figure 3:**
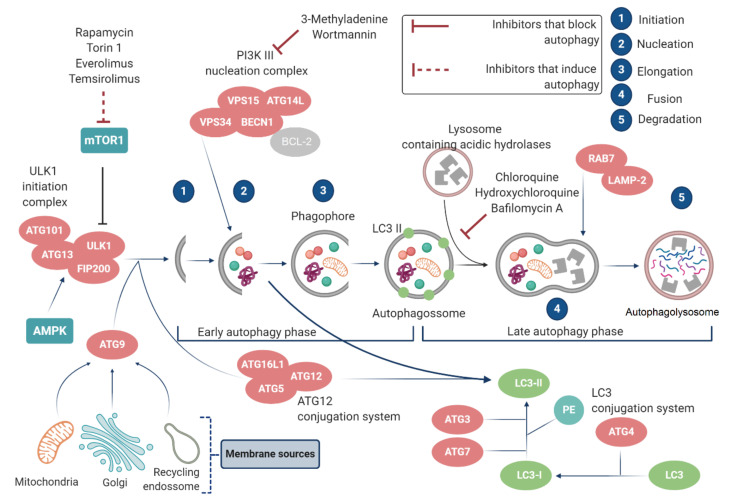
Steps of macroautophagy.

Macroautophagy, hereinafter called autophagy (Figure 3), involves the formation of a transient double-membrane structure, the phagophore, which encloses and isolates the cytoplasmic components to form the autophagosome. When the double-membrane autophagosome matures, it fuses with the lysosome to form the autophagolysosome to degrade its content and recycle macromolecules for reuse [56,57]. Although all three types of autophagy are different from each other on the basis of inducing signals, temporal aspects of induction, type of load, and sequestration mechanism, they culminate in (and strictly depend on) lysosomal degradation [58].

Autophagy can be a selective or non-selective lysosomal degradative process [59]. The selective autophagy involves the degradation of specific targets, e.g., protein aggregates (aggrephagy), lipids (lipophagy), glycogens (glycophagy), mitochondria (mitophagy), ribosome (ribophagy), peroxisomes (pexophagy), pathogens (xenophagy), endoplasmic reticulum (ER)-phagy, and nucleophagy (nucleus) [60,61,62]. On the other hand, in non-selective autophagy, the cytoplasmic contents are randomly sequestered and degraded [63].

### 2.2. Autophagy Regulation

#### 2.2.1. mTOR and ATGs

The autophagic mechanism can be summarized in different stages (or in five stages or phases): initiation, autophagosome nucleation, expansion and elongation of the autophagosome membrane, lysosome fusion, and degradation of intravesicular products [64,65]. This process is controlled by a series of proteins that function as complex and multiple signaling pathways [45,66]. In this context, ATG proteins are essential in these stages/phases and are involved in the regulation of autophagy [67,68].

The mammalian target of rapamycin (mTOR) is also an important protein complex in inducing autophagy. mTOR has two functional and biochemically distinct complexes, mTORC1 and mTORC2, both involved in the control of cell growth, survival, and proliferation. These complexes are serine/threonine kinases related to the activity of multiple kinases, including phosphatidylinositol 3-kinase (PI3K) that in general acts in response to growth factors, signs of stress, and nutritional status. AKT, a serine/threonine protein kinase also known as protein kinase B, is one of the main molecules downstream of PI3K. Thus, the PI3k/AKT/mTOR pathway has a strong influence on the coordination of the balance between cell growth and death according to the cell condition [69].

mTORC1 is a protein complex that plays a key regulatory role controlling the balance between cell growth and catabolism, via autophagy, in response to nutritional status and a variety of stress signals [70]. mTORC1 interacts with unc-51-like autophagy-activating kinase 1 (ULK1) by inhibiting its activation through the phosphorylation of ULK1 Ser 757 and interrupting its interaction with AMPK, when AMPK is activated due to a lack of glucose, mTORC1 is inhibited, thereby decreasing the phosphorylation of ULK1 Ser 757, leading to the ULK1-AMPK interaction, AMPK then phosphorylates ULK1 in Ser 317 and Ser 777, activating it and eventually inducing autophagy [71]. Furthermore, ULK1 forms a tetrameric complex with ATG13, ATG101 and FIP200, necessary for the recruitment of VPS34 complex for phagophore [72]. VPS34 complex is part of the PI3K class III complex (PIK3C3), composed of ATG14, VPS15 (PIK3R4) and beclin-1 [73]. The class III complex of PI3K produces phosphatidylinositol 3-phosphate (PI(3)P) at the phagophore initiation sites in the omegasome (a particular subdomain of the ER membrane that consists of a lipid bilayer membrane enriched for PI(3)P) [74,75].

The family of the ubiquitin-type light chain 3 (LC3; the mammalian ortholog of ATG8) protein is also involved in the formation of autophagosome. The microtubule-associated protein LC3 is translocated from the cytosol to the isolation membrane of the nascent autophagosome [76,77]. ATG4, a cysteine protease, is responsible for the proteolytic cleavage of pro-LC3, generating LC3-I [78,79]. ATG7, an E1-like enzyme, is necessary for the conjugation of ATG12 to ATG5 that interacts with ATG16L (an ortholog of ATG16 in yeast) in a non-covalent manner to form the ATG12/ATG5/ATG16L complex. The ATG5/ATG12/ATG16L complex, located at pre-autophagosomal structure (PAS), acts as an E3-like enzyme, stimulating the binding of LC3-I to phosphatidylethanolamine (PE) to form lipidized LC3 (LC3-II) [80,81,82]. In parallel, the conjugation of LC3-I with PE also occurs in a mechanism dependent on ATG7 and ATG3 [83,84].

LC3-II is found in the inner and outer membranes of the autophagosome and interacts with p62 to promote selective uptake and load degradation [78]. In addition to autophagosome maturation, LC3-II is also involved in the extension and closure of autophagosome membrane [79,85]. On the other hand, the multifunctional p62 protein is a vital component of the autophagy mechanism involved in protein quality control. p62 acts as an adapter molecule, binding to the charge by non-covalent interaction with ubiquitinated or polyubiquitinated residues and promoting its proteasomal degradation [86,87].

The next step for maturation in this self-degrading process is the fusion of the autophagosome with the lysosome to provide its content for degradation. The degradation of the autophagosome’s internal membrane occurs in a LC3-dependent manner [57]. The organelle that forms after the fusion of autophagosome and lysosome is generally referred as autophagolysosome [88]. The molecular mechanisms of autophagosome–lysosome fusion are mediated by fusion proteins, including Qbc-SNARE synaptosome associated protein 29 (SNAP29), Qa-SNARE syntaxin17 (STX17), lysosomal R-SNARE vesicle associated membrane protein 8 (VAMP8), small guanosine triphosphatase (GTPase) RAB7, homotypic fusion and protein sorting (HOPS) tethering complex, and accessory proteins such as ATG14 [89,90,91,92].

The degradation of intravesicular products is the last stage of the autophagic process. The acid hydrolases in the lysosome degrade the autophagic charge and the degrading products are recycled and released back to the cytosol [93]. Cathepsins, lysosomal proteases, are also involved in this process, leading to proteolytic degradation within the lysosome [94].

In particular, LC3-II and p62 are widely used as autophagic markers. While LC3-II is an autophagosome marker, p62 is a substrate for autophagy. As the synthesis and processing of LC3 increases during autophagy and the amount of LC3-II reflects the number of autophagosomes, LC3-II is generally used to measure the autophagic process [95]. p62 is used to monitor autophagic flux, as it is degraded by autophagy, that is, in autophagic suppression an increase in p62 levels occurs and autophagic activation correlates with a decrease in p62 levels [96,97]. Although the measurement of p62 levels are useful, to avoid undue correlations (because p62 acts in other pathways and interacts with other molecules not related to autophagy), it is recommended that the monitoring of autophagic flux be analyzed in combination with other methods, such as LC3-II turnover [98].

#### 2.2.2. p53

**Figure 4:**
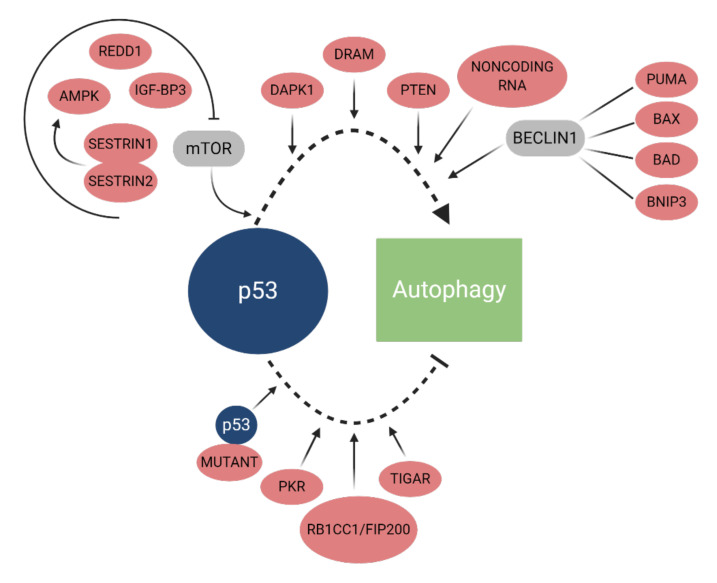
Regulation of autophagy by p53 signaling.

The autophagic cascade can also be regulated by p53 (Figure 4), which can act as an activator or inhibitor, depending on its subcellular location, either the cytoplasm or the nucleus [99]. Although nuclear p53 causes pro-autophagic responses that depends on the transcriptional activity of p53, cytoplasmic p53 induces anti-autophagic effects in a transcriptionally independent manner [100,101]. Under physiological conditions, p53 located in the intranucleolar regions is ubiquitinated by human double minute 2 (HDM2) protein and then transported to the cytoplasm for proteasomal degradation. HDM2, an ubiquitin ligase of p53, is a key regulator of p53 protein that controls p53 levels. This process inhibits p53-mediated transactivation and therefore inhibits autophagy [102,103].

p53 plays an important regulatory role in the autophagy process through different molecular mechanisms, such as inhibition of the mTOR signaling pathway, positive regulation of target genes, and interaction with autophagic proteins [101,104]. p53 induces autophagy by inducing expression of AMP-activated protein kinase (AMPK), regulated in development and DNA damage responses 1 (REDD1), and insulin-like growth factor-binding protein 3 (IGF-BP3), suppressing mTOR [105,106]. The proteins Sestrin1 and Sestrin2 are activators of AMPK that, under genotoxic stress, induce positive regulation of dependent p53, which has been shown to contribute to the induction of autophagy by inhibiting mTOR [107].

Alternatively, the positive regulation of target genes downstream of p53, such as death-associated protein kinase 1 (DAPK1), the damage-regulated autophagy modulator (DRAM), and phosphatase and tensin homolog deleted on chromosome 10 (PTEN), is also able to initiate autophagy [104,108,109]. In addition, pro-apoptotic proteins from the BCL-2 family (PUMA, BAX, BCL-2 associated agonist of cell death (BAD), and BCL-2 interacting protein 3 (BNIP3) can be directly transacted by p53 and further induce autophagy through interaction with beclin-1 [110,111]. BCL-2 has been indicated as a key molecule in autophagy. Interestingly, ER BCL-2 negatively regulates autophagy mediated by beclin-1, while mitochondrial BCL-2 regulates an antiapoptotic effect [112,113,114].

Under physiological conditions, p53 inhibits autophagy by binding and inhibiting RB1 inducible coiled-coil 1 (RB1CC1, also known as FIP200 or ATG17) and activating protein kinase RNA-activated (PKR). The regulation of autophagy by the RB1CC1/FIP200 p53-dependent manner affects the levels of stability and phosphorylation of ULK1 through regulatory interactions with mTOR signaling. Alternatively, p53 can inhibit autophagy by activating PKR in response to intracellular accumulation of double-strand RNA [115,116]. Autophagy can also be inhibited by the positive regulation of tumor protein P53 (TP53)-induced glycolysis and apoptosis regulator (TIGAR). As a target gene downstream of p53, TIGAR inhibits autophagy, promoting the pentose phosphate pathway and reducing reactive oxygen species (ROS) formation [117,118,119].

#### 2.2.3. Non-Coding RNAs

Long non-coding RNAs (lncRNAs) and microRNAs (miRNAs) can also regulate cell autophagy by modulating gene expression [120]. Fan et. al. [121] reported that MiR-18a upregulation caused autophagic activation in triple negative cancer cells by inhibiting of mTOR. Moreover, miR-24-3p enhanced cell proliferation, migration, invasion, and autophagy and reduced apoptotic cell death by suppressing the death effector domain containing (DEDD) and p62 activation in bladder cancer [122]. Seca et. al. [123] demonstrated upregulation of MiR-21 in chronic myeloid leukemia, as well as treatment with anti-miR-21 augmented beclin-1, Vps3, and LC3-II. The level of B-Raf proto-oncogene (BRAF)-activated lncRNA (BANCR) was found to be higher in papillary thyroid carcinoma than in counterpart tissue and was involved in cell proliferation and autophagic activation [124]. Likewise, the expression level of LncRNA highly upregulated in liver cancer (HULC) was higher in ovarian epithelial carcinoma than in normal samples and was seen to be the cause of tumorigenesis by inhibiting ATG7 [125].

## 3. Dual Role of Autophagy in Cancer

Autophagy has been related in different context-dependent roles in cancer. Before the beginning of tumorigenesis, in healthy tissues, autophagy is necessary for genomic stability, as a cytoprotective mechanism to prevent mutations, preventing genotoxicity from the production of reactive oxygen species (ROS) by the mitochondria and malignant transformations necessary for the appearance of cancer [126,127]. However, if carcinogenesis is already established, autophagy can be useful for tumor survival, allowing cells to reprogram their metabolism.

Autophagy is also found lower in some cancer tissues compared with their normal counterparts, indicating that autophagy can acts as a tumor suppressive [128]. Autophagy is important for the suppression of spontaneous tumorigenesis [127], e.g., as mentioned above, mice with systemic mosaic deletion of atg5 and liver-specific atg7^−^/^−^ develop benign liver adenomas [129]. Moreover, beclin-1 acts as both autophagy mediator and tumor suppressor [25]. Interestingly, others tumor suppressors, e.g., Bax-interacting factor-1 (BIF-1) and PTEN, induce autophagy, and some oncogenes, e.g., BCL-2 and extracellular signal-regulated kinase (ERK), inhibit the autophagy process [130,131,132,133]. The role of autophagy in tumor suppression has been explained in at least three mechanisms: inhibition of necrosis-mediated inflammation, maintenance of genome integrity, and autophagy-mediated cell death and senescence [134].

A crosstalk between autophagy and epithelial–mesenchymal transition (EMT) is also observed with dual action. Interestingly, EMT can induce or inhibit autophagy, and autophagy can also trigger or repress EMT [135]. In renal cell carcinoma cells, EMT-like phenotype displays a higher autophagy flux, and the use of an autophagy inhibitor was associated with suppression of EMT, indicating that autophagy inhibition is a target to eliminate EMT [136]. On the other hand, genetic inhibition of ATG5, ATG7, or Beclin-1 cause augmentation of cell motility and invasiveness along with upregulation of the transcription factors SNAIL and SLUG in glioblastoma cells, indicating that autophagy induction can also be a target to inhibit EMT [137].

**Figure 5:**
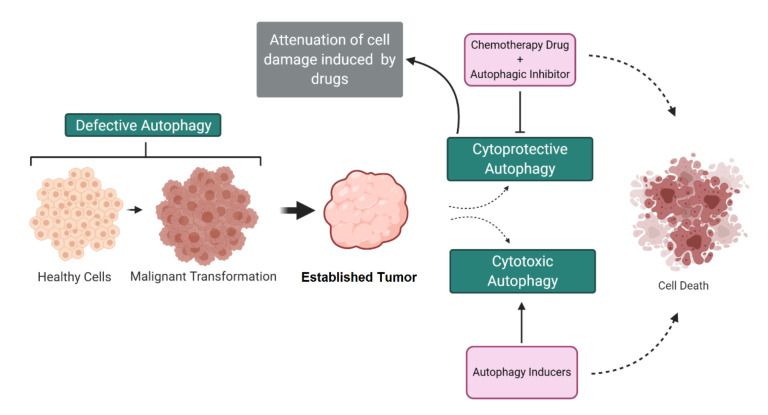
Different roles of autophagy in cancer.

The metabolites generated by autophagy can energetically supply tumor cells for growth and survival in metabolically hostile microenvironments, such as chemotherapy [138,139]. Under these conditions, autophagy acts as a cytoprotective mechanism, increasing tumor aggressiveness and drug resistance [4,138,140]. In cancer cells controlled by Ras, autophagy plays an important role in cell survival under conditions of metabolic stress [141,142]. Kirsten rat sarcoma (KRAS)-driven cancer shows high levels of autophagy in order to sustain its metabolism. In particular, genetic inhibition of ATG5 or ATG7 delays spontaneous lung and pancreatic KRAS-driven tumorigenesis in genetically modified mouse models [143,144]. In these situations of cytoprotective autophagy, the association of autophagic inhibitors and other chemotherapeutic drugs has been used successfully to reduce tumor growth [145]. Finally, autophagy machinery can also be recruited by anticancer drugs to mediate programmed cell death. This is an interesting therapeutic strategy, used mainly in cancers that are resistant to apoptosis, such as cancer cells with p53 deficiency [4,94]. Figure 5 presents the different roles of autophagy in cancer.

## 4. Autophagy-Mediated Resistance in Cancer Cells

Currently, most of anticancer drugs induce apoptosis as a mechanism of cell death. However, inactivation of the apoptotic pathway and the development of resistance to multiple drugs are inevitable after prolonged exposure to chemotherapeutic agents. In tumor samples from patients with colorectal cancer, the expression of the glycoprotein P (P-gp) was found to be positively correlated with the expression of the autophagy markers LC3 and beclin-1 [146]. In this context, autophagic inhibitors, such as chloroquine (CQ) and hydroxychloroquine (HCQ), have been extensively tested as agents that are capable of increasing cell death in chemoresistant tumors and have shown promising results [140,147,148]. CQ and HCQ are among the U.S. Food and Drug Administration (FDA)-approved autophagy inhibitors for humans, and several clinical cancer trials with these drugs have been conducted. However, side effects and low effectiveness limit their clinical application. For this reason, new autophagy inhibitors need to be investigated.

Analyzing the mechanism of action of BCL-2 homology domain-3 (BH-3)-mimetic obatoclax (also known as GX15-070) in colorectal cancer cell lines HT29 and SW480, it inhibited autophagy flux in late stage in a starvation situation. Obatoclax induced cell death independent of beclin-1, ATG7, and ATG12, important molecules in the early stages of autophagosome formation [149]. Similar results were found using cisplatin resistance and sensitive human esophageal cancer cell lines (obtained by prolonged culture of parental cell lines with sublethal concentrations of cisplatin). Yu et. al. [150] observed that obatoclax directly compromises lysosomal function, blocking the autophagic flux due to loss of cell viability. This study also reports that obatoclax blocks the flux of autophagy in the late degradation stage, similar to CQ [150]. Here, we discussed some chemotherapy drugs that can induce autophagy-mediated resistance in cancer cells.

### 4.1. Cisplatin and Paclitaxel

In ovarian cancer, acquired resistance occurs in most patients treated with cisplatin, a DNA intercalator, and paclitaxel, a microtubule stabilizer, first-line chemotherapeutic agents for various types of cancer [151]. However, inhibition of autophagy may improve cisplatin-induced apoptosis in some chemoresistant cells [152]. In that context, treatment with cisplatin induces resistance related to autophagy in ovarian cancer, increasing the levels of LC3-II and the degradation of p62 and activating mitogen-activated protein kinase (MAPK) pathways (that mainly include extracellular signal-regulated kinase—ERK, p38, and c-Jun N-terminal kinase—JNK), with ERK signaling fundamental to the mechanism of action. Furthermore, knockdown of ERK and pharmacological inhibition decreases LC3 levels and increases cisplatin-induced cell death. Similarly, ATG5 knockdown increases cisplatin-induced apoptosis [152].

Resistance linked to paclitaxel, the first-line chemotherapy against ovarian cancer, appears to be related to increased levels of thioredoxin domain-containing protein 17 (TXNDC17), a ubiquitous cytosolic protein that acts as a chemoresistant promoter and activates cytoprotective autophagy [153]. Therefore, the combination of paclitaxel with a late autophagic flux inhibitor can overcome resistance to the treatment of urothelial cancer cells [154]. In paclitaxel-resistant cells of bladder cancer, the combination of obatoclax and paclitaxel sensitizes cells to apoptotic cell death by blocking the autophagic flux [154].

### 4.2. Tyrosine Kinase Inhibitors

Epidermal growth factor receptor (EGFR) is an important tyrosine kinase receptor upstream of the PI3K/AKT/mTOR pathway. Thus, EGFR is a negative regulator of autophagy. Therefore, tyrosine kinase inhibitors, such as the chemotherapies gefitinib, erlotinib, lapatinib and neratinib, induce autophagy in different types of cancer [155,156,157]. Moreover, Wei et. al. [158] demonstrated that active EGFR binds to beclin-1, inhibiting its autophagy function in non-small cell lung cancer (NSCLC), and EGFR-mutated NSCLC cells presented improved tumor progression and partial tumor resistance to therapy with tyrosine kinase inhibitors.

Gefitinib has been recommended for the vast majority of NSCLC; however, chemotherapy resistance is common in the first year of treatment and constitutes the main factor of recurrence [159,160]. In vitro and in vivo studies show that autophagy can cause resistance to gefitinib in NSCLC cells; ERK phosphorylation promotes autophagy, stimulating the formation of LC3B-II and degradation of p62. Thus, suppression of autophagy or ERK inhibition reverses resistance to gefitinib [161].

In addition, the synergistic effects of gefitinib plus resveratrol also represent a strategy to overcome acquired resistance to treatment. Resveratrol plus gefitinib inhibits the proliferation of gefitinib-resistant NSCLC cells, increasing the intracellular concentration of gefitinib and blocking its elimination. However, co-treatment significantly induced autophagy with increased levels of LC3B-II and a high number of cells labeled with monodansylcadaverine (MDC), an autophagic vacuole marker. Apoptotic cell death has also been observed, and the inhibition of autophagy with 3-methyladenine (3-MA, an early-stage autophagy inhibitor that blocks the formation of autophagosome by inhibiting PI3K) has increased apoptosis and revoked senescence [162]. Resveratrol-induced apoptosis was also increased by pharmacological and genetic inhibition of autophagy in esophageal squamous cell carcinoma [163].

Erlotinib exposure is also capable of stimulating autophagy in lung adenocarcinoma and the inhibition of autophagy by CQ or silencing of ATG5 or beclin-1, increases sensitivity to erlotinib [164]. Moreover, the presence of protective autophagy is also observed in HER2-positive breast cancer cells treated with lapatinib [165]. On the other hand, neratinib degrades serine/threonine protein kinase (MST40) through autophagy as part of its cytotoxicity mechanism in pancreatic cancer cells [166].

Sorafenib, an approved multikinase inhibitor for patients with advanced stage of hepatocellular carcinoma (HCC), acts as an autophagy inducer by inhibiting mTORC1 [167] or activating AKT pathway [168], causing cell death or survival [169]. In HCC, the inhibition of autophagy potentiated the antitumor effect in a xenograft model in nude mice with Huh7 cells [167].

In a recent study, Liu et. al. [170] observed that gankirin (also known as PSMD10 or p28GANK) promoted tumorigenesis, metastasis, and drug resistance via activation of β-catenin/cellular myelocytomatosis (c-MYC) in human HCC. In another study by Luo et. al. [171], the authors observed that gankyrin is often overexpressed in HCC and promotes autophagy as a survival mechanism in cells treated with sorafenib. Gankirin-mediated resistance to sorafenib was completely blocked by the knockdown of ATG7, CQ, or 3-MA. Furthermore, in the analysis of human HCC biopsies, high levels of gankyrin and ATG7 were predictors of poor prognosis. Therefore, gankyrin can be a potential molecular target to overcome the resistance of sorafenib-treated HCC cells [171].

### 4.3. Trametinib

Interesting strategies have emerged to circumvent the current poor prognosis in pancreatic ductal adenocarcinoma (PDAC), for which autophagy plays a cytoprotective role in response to chemotherapy [172]. Some studies have shown that the survival of cancer cells controlled by Ras requires autophagy to maintain survival in conditions of metabolic stress and therefore has a high basal rate of autophagy [141,142]. Trametinib, a MAPK/ERK kinase (MEK) inhibitor drug, inhibits KRAS/RAF/MEK/ERK signaling, leading to a protective autophagic response [141]. Activation of key molecules in the modulation of autophagy, such as the LKB1/AMPK/ULK1 pathway, protects PDAC cells from the cytotoxic effects of trametinib. Thus, blocking the autophagic pathway in patients with PDAC with the combination of trametinib plus HCQ may represent a new treatment strategy [141]. Consistent with these data, Bryant collaborators [142] demonstrated that inhibition of MEK/ERK signaling induced autophagic flux, also as a mechanism of metabolic adaptation. Thus, synergistic inhibition of autophagy and ERK/KRAS signaling decreases glycolytic function and PDAC proliferation. Therefore, the combination of MEK/ERK inhibition and autophagy block may be effective in mediating antitumor activity in KRAS-controlled PDAC.

### 4.4. Gemcitabine

Gemcitabine, an antimetabolite chemotherapeutic drug, has been also reported to induce cytoprotective autophagy. Preoperative inhibition of autophagy with HCQ plus gemcitabine has also been tested in patients with pancreatic adenocarcinoma. Gemcitabine and HCQ were administered for 31 consecutive days until the day of surgery and proved to be an effective, safe, and well-tolerated option [173].

In a phase II randomized clinical trial, 112 patients with previously untreated metastasis or advanced PDCA were divided into two groups: in group 1, patients received gemcitabine, HCQ, and albumin-bound (NAB)-paclitaxel; in group 2, only gemcitabine and NAB-paclitaxel were received by the patients. The objective was to determine whether the association with HCQ would improve overall survival after 12 months. The results showed that the association improved the response rate but did not improve overall survival after 12 months. However, the lack of genomic data for most patients limited the conclusions on the basis of the status of the KRAS mutation [174]. Wild-type KRAS patients had a higher overall survival rate, regardless of the stage of diagnosis and chemotherapy regimen [175].

Gemcitabine also induces autophagy in lung cancer cells [176] and mTOR-independent autophagy in human triple-negative breast cancer cells. 3-MA increases the sensitivity of these cells to treatment [177].

### 4.5. 5-Fluorouracil

The treatment of advanced colorectal cancer has been carried out by the chemotherapist 5-fluorouracil (5-FU) an antimetabolite chemotherapeutic drug for many decades [178]. However, in vitro studies in human colon carcinoma cell lines HCT116 and HT29 and in vivo experiments in tumor tissues of xenograft mice demonstrate that autophagy is activated as a survival mechanism in cells treated with 5-FU. Therefore, a growing research effort is dedicated to sensitizing colorectal cancer to treatment with 5-FU through the modulation of autophagy, a fundamental mechanism in the development of resistance to treatment with 5-FU [139].

Curcumin impairs the autophagic flux, as it acts as a specific chemosensitizer for cancer and, in association with 5-FU, decreases AMPK/ULK1-dependent autophagy, modulates AKT, and increases apoptosis, amplifying the antitumor effects of 5-FU in colon cancer cells [145] and in human melanoma cells [179]. Li et. al. [180] also demonstrated that 3-MA inhibitor increases the cytotoxicity of 5-FU in HT29 colon cancer cells.

### 4.6. Cytarabine

Cytarabine (AraC) is an antimetabolite drug that acts by interfering with the synthesis of DNA and RNA. It is effective for hematological neoplasms, such as acute myeloid leukemia (AML), but in subsequent therapies, most patients have resistant disease [181]. The expression of chemokine type 4 receptor (CXCR4) on the cell surface of several cancer cells is related to cell proliferation and migration [182]. In AML, CXCR4 is correlated with poor prognosis, relapse, and low overall survival rates [183]. Activation of CXCR4 signaling in AML cells increases autophagic activity and cell survival, leads to drug resistance and decreases AraC-induced apoptosis [184]. AraC induces mTOR-dependent cytoprotective autophagy and modulates AKT, AMPK and ERK pathways in leukemic cell lines (HL-60 and MOLT-4) and in primary leukemic cells. Pharmacological and genetic inhibitors of autophagy increase AraC cytotoxicity, leading to mitochondrial depolarization, oxidative stress, caspase activation, and apoptotic cell death [185].

Leukemia-initiating cells (LICs) are a subset of self-renewable cells related to drug resistance. AraC treatment activates autophagy in LICs. In the absence of ATG7, AraC effect is enhanced [186]. The inhibition of autophagy by 3-MA increased cell death in HL-60 leukemia cells treated with AraC, but the use of CQ did not have the same effect [187].

### 4.7. Tamoxifen

The anticancer drug tamoxifen, an estrogen receptor antagonist, widely used for the treatment of ER-positive breast cancer, is associated with high rates of resistance related to high level of metastasis-associated 1 (MTA1), an autophagic regulator, also associated with tumorigenesis and breast metastases [188,189,190]. Interestingly, the genetic inhibition of ATG7, ATG5, or beclin-1 by small interfering RNA (siRNA) reverses the acquired resistance against tamoxifen [191].

Tamoxifen acts by inhibiting the enzymes involved in the final stages of cholesterol biosynthesis, called cholesterol epoxide hydrolase (ChEH). Its action triggers the production and accumulation of cholesterol-5,6-epoxide metabolites responsible for cell differentiation and death, which triggers the survival of autophagy to limit the effectiveness of tamoxifen [192].

In addition, Das et. al. [193] observed that the resistance acquired by tamoxifen is related to reduced ATP production and high expression of glycolytic molecules, including lactate dehydrogenase A (LDHA) that catalyzes the interconversion of pyruvate and L-lactate with concomitant interconversion of NADH and NAD^+^ [194]. LDHA is found in human cancers at higher levels compared to normal tissues and plays an important role in the development, invasion, and metastasis of some types of cancer. In particular, LDHA interacts with beclin-1 to induce pro-survival autophagy. The inhibition of LDHA reduces pro-survival autophagy, with restoration of apoptosis, overcoming resistance to tamoxifen in breast cancer (resistant MCF-7 cells and resistant T47D cells). Consequently, LDHA inhibitors may be good strategies for overcoming the resistance acquired by tamoxifen [193]. Moreover, blocking LDHA by FX11 (a potent LDHA inhibitor) decreases ATP levels and induces significant oxidative stress, leading to cell death, prevented by the antioxidant N-acetylcysteine [195].

### 4.8. Temsirolimus and Everolimus

In renal cell carcinoma cell lines, the rapamycin analogue temsirolimus induced cell death with synergistic effects in combination with HCQ in culture and in an orthotropic mouse model [196]. Furthermore, in patients with advanced solid tumors and melanoma, temsirolimus plus HCQ demonstrated the safety and preliminary benefits in a phase I clinical trial [197].

Everolimus (also known as RAD001) is an mTOR inhibitor approved by the FDA for the treatment of advanced clear cell renal cell carcinoma (ccRCC) because it demonstrated efficacy at 10 mg daily versus placebo-treated patients. The mean progression-free survival (PFS) of 4.01 months was seen in patients treated with everolimus, compared with 1.87 months in patients treated with placebo [198]. mTOR inhibition induces autophagy that may act as a resistance mechanism. The association of HCQ 600 mg twice daily and 10 mg daily everolimus induced a 6-month PFS rate in 40% of the RCC patients [199].

These data corroborate that autophagy can protect cancer cells from apoptosis, preventing damage and promoting a resistance profile. Interestingly, cytoprotective autophagy may be due to some clinically useful chemotherapy drugs, including cisplantin, paclitaxel, gefitinib, trametinib, gemcitabine, 5-FU, sorafenib, cytarabine, tamoxifen, temsirolimus, and everolimus. Additional investigations should be carried out with these drugs to better understand how this effect can be avoided. In this context, autophagic inhibitors are great tools to overcome resistance to anticancer drugs.

## 5. Autophagy-Mediated Cell Death in Cancer Cells

Although, by definition, autophagy is a biological process of survival, autophagy is also reported as a mechanism of cell death. The Nomenclature Committee on Cell Death (NCCD) defines autophagy as a regulated cell death that depends mechanically on autophagic machinery or their components to cause cell death [66]. In this case, only studies where cell death can be rescued or prevented by chemical or genetic inhibition of autophagy can be attributed to cell death mediated by autophagy [8,200]. The involvement of at least two different proteins from the autophagic machinery is also recommended as a criterion to confirm induction of autophagy-mediated cell death, usually LC3-II (increase) and p62 (decrease). This type of cell death can occur due to excessive autophagy, which has a morphological and molecular process distinct from apoptotic or necrotic cell death and is independent of caspase [66,200].

Additionally, some international guidelines also define the roles of autophagy in the cell death process as (I) cell death associated with autophagy, when the induction of autophagy occurs together with the induction of apoptosis (or other type of cell death)—in this case, autophagy has no active role in cell death, it is only present during the cell death process; (II) cell death mediated by autophagy, in which the induction of autophagy causes apoptosis; and (III) autophagy-dependent cell death, a distinct mechanism of cell death that occurs independently of apoptosis or necrosis (similar to the definition of the NCCD) [201,202]. Moreover, as mentioned above, autophagy machinery also participates in the cell death by autosis [11]. In this section, we discuss some drugs that can cause autophagy-mediated cell death as defined by NCCD.

### 5.1. Sorafenib

Although gankirin-mediated resistance to sorafenib was completely blocked by the knockdown of ATG7, CQ, or 3-MA in HCC cells, indicating that autophagy is a mechanism associated with sorafenib resistance (discussed on Section 4), sorafenib induced an increase in LC3 lipidation and p62 degradation triggered by AKT inhibition in a MAPK-independent autophagy-mediated cell death in ACHN and 786-O renal carcinoma cell lines. The knockdown of ATG5 or autophagy inhibitor 3-MA promoted an increase in viable cells and resistance to sorafenib [203]. These data indicate that sorafenib can induce autophagy in both cytoprotective and cytotoxic ways in different contexts.

### 5.2. Itraconazole

The repositioning of drugs consists of the use of drugs already used in the clinic for a new therapeutic indication, with the advantage of less financial investment and greater security [204]. Many of these drugs, such as clarithromycin [205], tioconazole [206], and itraconazole [207,208,209], have been relocated to anticancer therapy because they induce autophagy inhibition or cause autophagy-mediated cell death [208,210].

The antitumor action of antifungal itraconazole, an inhibitor of ergosterol synthesis, has been associated with autophagy due to inhibitory effects on cell proliferation and inhibition of mTOR and Hedgehog (HH) pathways [207,208,209]. Itraconazole has cytotoxic effects related to autophagy in different cancer cells, including breast cancer cell lines [208] and glioblastomas [211].

Wang et. al. [208] demonstrated that itraconazole has cytotoxic effects on breast cancer cell lines MCF-7 and SKBR-3 through mitochondrial depolarization followed by apoptosis dependent on caspase-3 activation. Moreover, itraconazole also induced cell death mediated by autophagy by increasing LC3-II and degradation of p62, regulated by pharmacological inhibition of HH pathway. The autophagy-mediated cell death was confirmed by the specific small interfering RNA (siRNA) of ATG5 and autophagy inhibitor 3-MA, whose cell death induced by itraconazole was partially inhibited in breast cancer cells.

### 5.3. Clozapine

Another drug in repositioning is clozapine, which induces autophagy via AMPK/ULK1/Beclin-1 signaling pathway [212]. Clozapine is an atypical antipsychotic widely used in the treatment of patients with refractory schizophrenia. In a study of 59,257 patients with schizophrenia, this group was found to have a lower overall risk of cancer compared to the general population [213]; however, the prevalence of smoking among schizophrenic patients is approximately 70%, contributing to increased risk of developing cancers associated with tobacco such as lung cancer [214].

In this context, Yin et. al. [210] investigated the role of clozapine on autophagy in lung cancer cells A549 and H1299. In this study, clozapine increased the levels of LC3-II, inhibited cell proliferation, arrested cell cycle, regulated the levels of p21 and p27, and induced autophagy-mediated cell death. On the other hand, treatment with bafilomycin A1 (late autophagic inhibitor) and knockdown of ATG7 decreased clozapine-induced autophagy and had cytotoxic effects.

### 5.4. SU11274

In a model of human NSCLC cells, SU11274, a small inhibitor of the c-Met molecule, induced autophagy-mediated cell death through ERK-p53 activation and ERK-mediated BCL-2 phosphorylation. Inhibition of autophagy by 3-MA suppressed SU11274-induced cell death [215].

### 5.5. Temozolomide and Arsenic Trioxide

Glioblastomas are extremely aggressive and have a poor prognosis due to resistance to radiotherapy, surgery, apoptosis, and most chemotherapeutic agents with pro-apoptotic effects [216,217,218]. Glioblastoma cells are less likely to resist autophagy, and inhibition of mTOR induces the autophagic process [218]. The drugs temozolomide (TMZ), an alkylating agent, and arsenic trioxide (ATO), an inorganic antileukemic compound, are known to induce autophagy-mediated cell death in glioblastomas [219,220] and leukemia cells [221]. However, autophagy can also lead to the mechanism of cytoprotection in gliobastomas, and autophagic inhibitors can be used to sensitize these cells to chemotherapy [222]. Furthermore, inhibition of autophagy at different stages of autophagic flux can determine whether there will be a cytotoxic effect or induction of cytoprotective effect by inhibitors [219].

Glioblastoma multiforme cell lines U87 and U251 treated with ATO showed a reduction in cell viability and autophagy induction, and increased levels of cleaved caspase-3. Meanwhile, when treated with ATO plus CQ (late stage autophagic flux inhibitor), a higher level of cleaved caspase-3 was found, indicating that the association improves apoptosis. This result was confirmed by the increase in the level of p62 in the treatment of ATO plus CQ when compared to the control. However, when knockdown of beclin-1 or 3-MA (initial stage autophagic flux inhibitor) were used, the cytotoxic effect of ATO on glioblastomas was avoided. Therefore, inhibition of the final stages of autophagy, by blocking the fusion of the autophagy lysosome, increases the cytotoxicity induced by ATO, but the same does not occur when the block occurs in the early stages of autophagy [219]. The same was observed in the malignant glioma cell line U373-MG treated with TMZ. Blocking autophagy by 3-MA completely inhibits cytotoxic effects and the use of bafilomycin A1 increases the cytotoxic effects of TMZ [223].

Clinical trials were conducted to determine the maximum tolerated dose and effectiveness of HCQ in combination with TMZ and radiation in the treatment of newly diagnosed glioblastoma. HCQ was administered to 16 patients who received doses of 200 to 800 mg orally/daily. At 800 mg, one patient had sepsis and three had neutropenia and thrombocytopenia. Thus, HCQ 600 mg/day was considered the maximum safe dose in this combination. However, clinical trials have also shown that inhibition of autophagy with HCQ at 600 mg/day was not consistent and no improvement in overall survival was observed [222].

### 5.6. Natural Products

Another compound associated with autophagy-mediated cell death in cancer cells is betulinic acid (BetA), a plant-derived cytotoxic compound. In multiple myeloma cell lines, BetA activates protein phosphatase 2A (PP2A), which regulates apoptosis and autophagy-mediated cell death according to different levels of BCL-2 expression. BCL-2 overexpression attenuates apoptosis and activates autophagy-mediated cell death by LC3-II expression and degradation of p62. 3-MA treatment and knockdown of ATG5 efficiently inhibited autophagy-mediated cell death [224]. On the contrary, in cells with low levels of BCL-2, caspase-3 cleaves PP2A, inactivating AKT and promoting apoptosis [224]. Since overexpression of the anti-apoptotic BCL-2 family members is observed in most patients with multiple myeloma [225], PP2A represents a target molecule to increase the effects of the autophagy modulators chemotherapeutic agents used for myeloma. In contrast, Yang et. al. [226] reports that BetA induces apoptosis and blocks autophagic flux in human multiple myeloma cells in vitro.

Cannabinoids are found in *Cannabis sativa*, with Δ9 tetrahydrocannabinol (THC) being the main active component. THC has been found to have antitumor effects by cell death mediated by autophagy through inhibition of the AKT/mTOR pathway. In human glioma, THC induced autophagy-mediated cell death in three different tumor xenograft models and when it was administered intracranially in two patients. Moreover, genetic and pharmacological inhibitors of autophagy prevented THC-induced cell death in glioma cells [227]. Cannabinoids promote cell death mediated by autophagy in melanoma cells, and treatment with CQ or ATG7 knockdown prevented THC-induced autophagy-mediated cell death [228]. Cannabinoids still appear to be overcoming resistance to oxaliplatin in human colorectal cancer cells. This reduces proliferation and induces autophagy-mediated cell death, increasing ROS production and reducing superoxide dismutase 2 levels [229].

Berberine is an isoquinoline alkaloid found in *Coptis chinensis* that has been used in traditional Chinese and Indian medicine to treat infections, cancer, and other diseases [230,231,232]. Berberine causes a significant decrease in cell viability and activates autophagy in colon cancer cells HCT-116 and DLD1, and hepatocellular cancer cell HepG2, inhibiting the degradation of heat shock 70 kDa protein 5 (HSPA5) [233], an autophagy inducer in conditions of lack of glucose [234]. Berberine also strengthens the link between HSPA5 and PI3K, proteins related to the remodeling of the membrane for formation of autophagosome. Berberine increased LC3 and beclin-1 expression and decreased p62 levels. The autophagic inhibitor 3-MA and ATG5 and beclin-1 silencing reduced the cytotoxic effect of berberine [233].

Although curcumin can impair the autophagic flux (discussed in Section 4), the association of curcumin and berberine in breast cancer cells MCF-7 and MDA-MB-231 stimulates cell death mediated by autophagy by positive regulation of beclin-1 and phosphorylation of JNK. Inhibition of autophagy with CQ and 3-MA strongly reduces the cytotoxicity induced by this combination [235]. In contrast, in glioblastoma cells U251 and U87, berberine induces autophagy by inhibiting the AMPK/mTOR/ULK1 pathway. It reduces tumor growth in vivo, but the treatment is more effective when berberine is combined with CQ [236].

Curcumin also acts as a sensitizer that increases the cytotoxicity of gefitinib and overcomes resistance in NSCLC cells with wild-type EGFR and/or KRAS mutation by induction of cell death mediated by autophagy These combination treatment effect can be reversed by pharmacological autophagy inhibitors (Baf A1 or 3-MA) or knockdown of Beclin-1 or ATG7, as well by pan-caspase inhibitor (Z-VAD-FMK) [237].

On the other hand, curcumin suppresses the PI3K/AKT/mTOR pathway, inhibits cell viability, and induces cell apoptosis in gastric cancer cell lines BGC-823, SGC-7901, and MKN-28. In addition, curcumin induces autophagy by the conversion of LC3-I to LC3-II and increases levels of ATG proteins. Administration of autophagy inhibitor 3-MA significantly increases apoptotic cell death, indicating that curcumin treatment induces protective autophagy in human gastric cancer cells in vitro [238]. These discrepant data may be related to the concentration of curcumin, time of incubation and the type of cancer cells used.

A study with colon cancer cells HCT116 and HCT8 showed that cell proliferation was reduced after treatment with α-hederin, a monodemosodic triterpenoid saponin found in *Nigella sativa*, with activation of autophagy and mitochondrial apoptosis. The treatment with α-hederin also induced ROS production, and this process was mediated by activation of AMPK/mTOR signaling. When oxidative stress was blocked by NAC or autophagy was inhibited by 3-MA, cell death was prevented [239].

Neferin, a natural alkaloid of *Nelumbo nucifera*, has been shown to induce autophagy-mediated cell death in a panel of cancer cells containing cell lines resistant to apoptosis, activating the ryanodine receptor and ULK1/PERK/mTOR/AMPK signaling cascades. Interestingly, cell death was prevented when ATG7 was silenced by genetic inhibition [240].

Ginkgetin, a bioflavonoid originating from *Ginkgo biloba* leaves, induces autophagy in lung cancer cells A549. In vitro and in vivo experiments showed an increased level of LC3, formation of ROS, decrease of p62, and formation of double membrane structures. Inhibition of autophagy with 3-MA or CQ prevented ginkgetin-induced cytotoxicity, and rapamycin, an autophagy inducer, increased the cytotoxic effect of ginkgetin [241].

Piplartine, also known as piperlongumine, is a naturally occurring alkaloid amide found in *Piper* species that exhibits several important pharmacological activities. In particular, piplartine and its derivatives have been studied extensively due to their anticancer potential [242,243,244,245,246,247]. Wang et. al. [248] reported that cell death caused by piplartine in human osteosarcoma U2OS cells can be prevented by 3-MA or reduced in cells without the ATG5 gene. This also improves autophagy activity without blocking autophagy flux. The authors concluded that piplartine induces the activation of the p38 pathway by stress response induced by ROS, triggering cell death mediated by autophagy [248]. On the other hand, although piplartine can induce autophagy in A549/DTX lung cancer cells, 3-MA or beclin-1 and ATG5 siRNAs improved piplartine-induced apoptosis, suggesting that piplartine-induced autophagy in A549/DTX cells can protect these cells from apoptosis [249]. Likewise, piplartine causes autophagy in prostate, kidney, and breast cancer cell lines, reducing AKT/mTOR signaling through ROS accumulation; however, CQ increased piplartine-induced cell death in vitro and in vivo [250].

Kaempferol, a histone deacetylase inhibitor, is found in different fruits and vegetables [251]. It presents different mechanisms of action against cancer cell lines, including autophagy-mediated cell death in gastric cancer [252] and HCC cells [253]. Kaempferol activates AMPK, upregulates the degradation of beclin-1 and p62, and converts LC3-I into LC3-II in HCC cell lines (HepG2, Huh-7, BEL7402, and SMMC) and primary cells of HCC. Inhibition of AMPK or inhibition of autophagy by 3-MA or beclin-1 small hairpin RNA protected the HCC cell from kaempferol effect [253].

Likewise, Wang et. al. [254] reported that fangchinoline, an alkaloid of *Stephania tetrandra*, induces autophagy-mediated cell death though p53/sestrin2/AMPK signaling in human HCC cells.

Altogether, we observed that autophagy is also an important type of cell death in cancer cells. Here, we summarize some of the autophagy inducers that can cause cancer cell death, including itraconazole, clozapine, SU11274, temozolomide, arsenic trioxide, and some natural products. New studies with autophagy inducers should be carried out to increase knowledge about the action potential of these drugs in cancer therapy. The discovery of new inducers of autophagy and the understanding of the mechanisms of action of those already known can lead to the development of new approaches for the treatment of cancer.

## 6. Autophagy in Cancer Stem Cell Biology

Tumors are composed of a heterogeneous cell population, with subsets of cells exhibiting distinct tumorigenic capabilities. Cancer stem cells (CSCs) are a small subpopulation of cancer cells with increased renewal capacity that play roles in tumor growth, cell and molecular heterogeneity, differentiation, and metastasis [255,256]. CSCs were first identified in AML and, in recent decades, have been identified in several solid cancers and hematological neoplasms of myeloid and lymphoid origin. In general, accumulated mutations and new genetic changes quickly differentiate CSCs, giving rise to tumor heterogeneity [257].

Currently, CSCs have been associated with failure of antineoplastic treatment due to tumor recurrence and resistance to conventional anticancer therapies, such as chemotherapy and radiotherapy [258]. In addition, cellular plasticity, resting state, highly efficient DNA damage repair, unregulated cell cycle, high aldehyde dehydrogenase activity, and increased expression of drug transporters and other resistance genes have been reported as mechanisms underlying survival and maintenance of CSCs [259,260].

CSCs are characterized by the presence of specific cell surface markers such as CD133, CD117, CD34, CD44, and CD54, which correlate with a poor clinical prognosis of tumor. In terms of the molecular characteristics, CSCs exhibit expression patterns of genes involved in the regulation of stem cell self-renewal, such as proto-oncogenes; tumor suppressors; or expression of signaling pathway components involved in the control of cell cycle, growth, survival, and death [261,262].

The pluripotency of CSCs allows the maintenance of undifferentiated cells, as well as unlimited growth and division, whose autophagy plays a fundamental role [255,263,264]. Therefore, several autophagy modulators targeting CSCs are under pre-clinical and clinical investigation [257,262]. In general, understanding the molecular mechanisms of maintenance of autophagy-dependent CSCs can be an attractive target for cancer treatment.

Tumors induce autophagy to adapt to adverse conditions serving as a source of nutrients and survival, in addition to acting on cell differentiation, which is important for the spread of the disease. In this context, inhibition of autophagy can provide a strategy to overcome the resistance and progression of CSCs. The inhibition of autophagosome degradation does not affect cargo sequestration and autophagosome maturation or formation. Thus, inhibition of autophagy genes by small molecules induces cell death in CSCs. A new generation of autophagy inhibitors, such as SAR405 and MRT68921, targeting catalytic components of autophagosome biogenesis, including VPS34 complex and ULK1, is under investigation. These drugs are effective against the early stages of autophagy and can provide better results for controlling CSCs [257,265]. Here, we describe the effect of autophagy inducers or inhibitors in CSCs to discuss the role of autophagy in this sub-population of cancer cells.

### 6.1. Glioblastoma

Glioblastoma CSCs have been reported to be involved in tumorigenesis, tumor maintenance, and therapeutic resistance. The effects of a new therapeutic strategy to inhibit autophagy using siramesine, a lysosotropic destabilizing drug, have been evaluated in glioblastoma CSCs. The results, in vitro and in vivo, showed that siramesin caused the death of tumor cells and inhibited the migration of tumor cells [266]. The inhibition of autophagy also sensitizes cells for treatment with TMZ in glioblastoma CSCs [267].

In contrast, natural compounds, such as rapamycin, resveratrol, and curcumin, have been reported to be capable of inducing autophagy in glioblastoma CSCs. These compounds could suppress the proliferation, migration, invasion, and growth of CSCs by induction of autophagy. Likewise, nigericin has also been shown to induce autophagy, suppressing the proliferation of glioblastoma cells along with the inhibition of CSCs [268].

The suberoylanilide hydroxamic acid (SAHA), an inhibitor of histone deacetylase, induces autophagy through the negative regulation of the AKT/mTOR signaling, and inhibits cell proliferation, promoting apoptosis in glioblastoma CSCs. Moreover, cells treated with SAHA displayed augmented formation of intracellular acid vesicle organelles, increased LC3-II and beclin 1 levels, and decreased p62. In addition, SAHA reduced tumor growth and induced autophagy in vivo xenograft assays [269]. Wang et. al. [270] reported that dactolisib (NVP-BEZ235), a new dual inhibitor of PI3K/mTOR, increases the radiosensitivity of human glioma CSCs in vitro, activating autophagy.

Cheng et. al. [271] previously redirected thioridazine, an antipsychotic medication, as an anti-glioblastoma and anti-CSC agent. Thioridazine induces autophagy in glioblastoma cell lines and positively regulates AMPK activity. Furthermore, in vivo studies, thioridazine upregulated LC3-II levels in spheroids formed by human glioblastoma U87MG cells, suppressed the tumorigenesis, and induced autophagy. More recently, Chu et. al. [272] reported that thioridazine induces autophagy in glioblastoma cells, not only positively regulating AMPK activity, but also improving p62-mediated autophagy and apoptosis through Wnt/β-catenin signaling. In addition, treatment with thioridazine combined with TMZ increased LC3-II expression and caspase-3 activity [272]. Similar effects mediated by autophagy via Wnt/β-catenin signaling pathway were also observed in mouse embryonic carcinoma cell line P19 after treatment with bioflavonoid apigenin [273].

### 6.2. Liver and Pancreatic Cancers

Autophagy contributes to the survival of CD133+ liver CSCs in the hypoxic and nutrient-free tumor microenvironment. CQ significantly increased apoptosis, decreased clonogenic capacity, and impaired spheroid formation capacity of CD133+ cells. Additionally, an increased tumor-forming ability of liver CSCs in vivo can be inhibited by the administration of CQ [274]. In a cancer patient-derived xenograft model, Balic et. al. [275] demonstrated that CQ targets pancreatic CSCs by inhibiting CXCR4 and HH signaling.

### 6.3. Breast Cancer

Breast CSCs have an extensive capacity for self-renewal and contribute to metastasis and therapeutic resistance. In vitro study demonstrated that resveratrol induces autophagy in breast CSCs, suppressing the Wnt/β-catenin signaling pathway. Transmission electron microscopy analysis, green fluorescent protein (GFP)-LC3-II puncta formation test, and Western blot for LC3-II, beclin-1 and ATG7, showed that resveratrol induces autophagy in breast CSCs. Resveratrol significantly inhibits proliferation, suppresses Wnt/β-catenin signaling pathway, and decreases the percentage of breast CSC population. In a model using nonobese diabetic/severe combined immunodeficient (NOD/SCID) mice, resveratrol effectively inhibited the growth of xenograft tumors and reduced breast CSC population in tumor cells [9]. In addition, 6-shogaol can inhibit breast cancer cells and breast CSCs by modulating Notch signaling pathway and inducing autophagy-mediated cell death. Furthermore, combination of CQ and 6-shogaol decreased cell death [276].

Studies on breast CSCs isolated from cell lines MCF-7 and SUM159 showed that resveratrol induces autophagy via Wnt/β-catenin signaling pathway in vitro and in a xenograft model, effectively reducing the population of CSCs, causing cell death mediated by autophagy. The LC3-II, beclin-1, and ATG7 molecules were upregulated. The inhibition of autophagy by CQ or the overexpression of β-catenin attenuated the cytotoxic effects of resveratrol on CSCs [9]. Furthermore, resveratrol inhibited AKT pathway and AMPK signaling in cisplatin-resistant human cancer cells, leading to autophagic- and apoptotic-mediated cell death [277]. Resveratrol also induces cell death mediated by autophagy and apoptosis in cervical cancer cells [278] and in prostate cancer cells [279].

The effects of everolimus have been evaluated alone or in combination with docetaxel. Stem cells from primary breast cancer cells were resistant to standard docetaxel treatment; however, the combined treatment of everolimus and docetaxel can inhibit the growth of breast CSCs in vitro and in vivo [280]. The susceptibility of breast CSCs to buparlisib (NVP-BKM120), a new generation of specific PI3K inhibitor, when used individually or in combination with trastuzumab or everolimus, was also investigated. The authors concluded that the combination of NVP-BKM120 plus trastuzumab or everolimus synergistically inhibits the growth of breast CSCs in vivo [281].

Liang et. al. [282] suggested that CQ has anti-CSC effects and may be an effective complement to current carboplatin chemotherapy regimens. CQ effectively targets CSCs in triple-negative breast cancer, inhibiting autophagy and mitochondrial structural damage, and repairing damaged double-stranded DNA breaks. In addition, CQ effectively decreases the ability of these cells to metastasize in vitro and in a xenograft model.

### 6.4. Gastric, Lung, and Colon Cancers

Gastric CSCs are also related to poor prognosis after standard chemotherapy. Western blot analysis showed that the levels of LC3-II were markedly increased in CD44+/CD54+ gastric CSCs. Moreover, the proportion of LC3-II/LC3-I protein levels was higher in gastric CSCs than in non-CSCs. On the other hand, 5-FU and CQ exhibited an inhibitory effect on the cell viability of gastric CSCs, and their combination further increased these inhibitory effects [283].

The clinical treatment of NSCLC patients with cisplatin can lead to drug resistance. In a study by Hao et. al. [284], CQ reduced CD133+ NSCLC CSCs and enhanced cisplatin effect in A549 cells. Moreover, cisplatin plus CQ treatment presented reduced autophagy levels and enhanced tumor growth inhibition.

Torin-1, an mTOR inhibitor, inhibited the growth, motility, invasion, and survival of CSCs isolated from three human metastatic colorectal cancers. Torin-1 also affected the expression of markers for cell proliferation, angio/lymphogenesis, and stemness, and suppressed tumor growth in vivo along with reduction in vessel formation. In addition, torin-1 did not affect the survival of normal colon stem cells in vivo, suggesting its selectivity in relation to CSCs [285].

### 6.5. Acute Myeloid Leukemia

Dendrogenin A (DDA) showed tumor-suppressive properties in vitro and in vivo, triggering lethal autophagy in leukemia [286,287]. Furthermore, DDA was 100 times more efficient than treatment with AraC in reducing viability in the subpopulation of progenitor/leukemic stem cells (LSCs) in samples from patients with AML. It acts as an agonist of liver-X-receptor (LXR), a nuclear receptor that triggers lethal autophagy by inducing the transcriptional expression of the pro-autophagic factors nuclear receptor subfamily 4 group A member 1 (NR4A1, also known as Nur77), nuclear receptor subfamily 4 group A member 3 (NR4A3, also known as NOR1), LC3-II and transcription factor EB (TFEB), and also of the biogenesis of lysosomes, necessary for the formation of autophagolysosomes. The knockout of autophagic mediators ATG7, PI3P, and beclin-1, and pharmacological inhibitors of autophagy bafilomycin A1 or HCQ, inhibited the formation of LC3-II and the cell death induced by DDA [287].

Folkerts et. al. [288] explored whether the inhibition of autophagy can be used as a treatment strategy for p53 wild-type AML. AML CD34+ LSCs were more sensitive to HCQ autophagy inhibitor than normal bone marrow CD34+ cells, suggesting that targeting autophagy may provide new therapeutic options for the treatment of AML.

**Figure 6:**
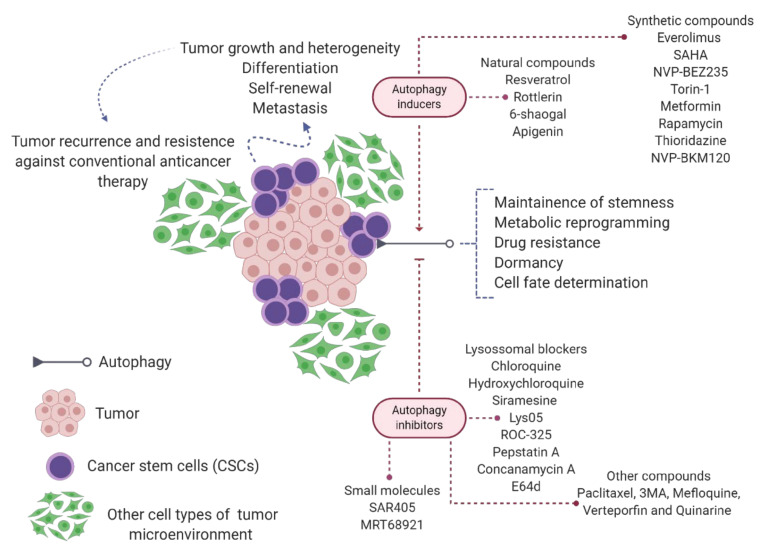
The role of autophagy in cancer stem cells.

Overall, these data reinforce that pharmacological approaches aimed at autophagy, alone or in combination with chemotherapeutic drugs, represent a promising strategy to combat CSC resistance to cancer therapy (Figure 6). Furthermore, understanding the mechanisms and molecular factors that regulate autophagy-mediated resistance in CSCs can improve our understanding to develop effective approaches to overcome aggression, drug resistance, and cancer recurrence in CSCs. Additionally, both autophagy inducers and inhibitors are able to eliminate CSCs.

## 7. Conclusions

Autophagy is a cell survival mechanism that in cancers plays an important role in the mechanisms of resistance to chemotherapy, especially in CSCs. Thus, the association of autophagy inhibitors with chemotherapeutic drugs can lead to the sensitization of these cells to chemotherapeutic drugs and, consequently, increases their effect. On the other hand, the excessive activation of autophagy can cause the cell to lose control of the activation of this survival mechanism and, consequently, lead to cell death mediated by autophagy. Thus, we have situations in which the autophagic inducers cause cell death and lead to reduced tumor growth. Consequently, both autophagic inhibitors and inducers are useful in the treatment of cancer. By this concept, autophagy is responsible, at least in part, for some cancer cells to be more resistant than others, which also depends on the cancer type. This biological variation can be also related to the amount of CSC subpopulation in each individual cancer patient that presents autophagy as an important survival mechanism. In this review, we summarized and discussed some of the inducers and inhibitors of autophagy in order to direct future research. Table 1 summarizes these autophagy modulator drugs. Further studies with these compounds must be carried out so that they can be used in clinical practice for the benefit of cancer patients.

## Figures and Tables

**Table 1:** Autophagic modulating drugs in neoplasms.

Drugs	Action	Number of Clinical Trial *
α-Hederin	Induces autophagy-mediated cell death in colorectal cancer cells [239]	-
6-Shogaol	Induces autophagy-mediated cell death in breast cancer cells [276]	-
Apigenin	Induces autophagy-mediated cell death in embryonic cancer cells [273]	1
Arsenic trioxide	Induces autophagy-mediated cell death in glioblastoma cells [220]	125
Berberine	Induces autophagy-mediated cell death in colon and liver cancer cells [233]	8
	Induces autophagy-mediated cell death in breast cancer cells [235]	
Betulinic acid	Induces autophagy-mediated cell death in myeloma cells [224]	1
Cannabinoid	Induces autophagy-mediated cell death in glioma [227]	22
	Induces autophagy-mediated cell death in melanoma cells [228]	
	Induces autophagy-mediated cell death in colorectal cancer cells [229]	
Chloroquine	Inhibits cytoprotective autophagy in liver cancer cells [274]	23
	Inhibits cytoprotective autophagy in breast cancer cells [282]	
	Inhibits cytoprotective autophagy in gastric cancer cells [283]	
	Inhibits cytoprotective autophagy in glioma [289]	
Clarithromycin	Inhibits cytoprotective autophagy in colorectal cancer cells [205]	60
Clozapine	Induces autophagy-mediated cell death in lung cancer cells [210]	2
Curcumin	Inhibits cytoprotective autophagy in colon cancer cells [145]	68
	Inhibits cytoprotective autophagy in melanoma cells [179]	
Dendrogenin A	Induces autophagy-mediated cell death in acute myeloid leukemia and melanoma [287]	2
Everolimus	Induces autophagy-mediated cell death in breast cancer cells [280]	604
Fangchinoline	Induces autophagy-mediated cell death in liver cancer cells [254]	-
Ginkgetin	Induces autophagy-mediated cell death in lung cancer cells [241]	-
Obatoclax (GX15-070)	Inhibits cytoprotective autophagy in colorectal cancer cells [149]	19
	Inhibits cytoprotective autophagy in esophageal bladder cancer [150]	
Hydroxychloroquine	Inhibits cytoprotective autophagy in acute myeloid leukemia [288]	81
Itraconazole	Induces autophagy-mediated cell death in breast cancer cells [208]	56
Kaempferol	Induces autophagy-mediated cell death in gastric cancer cells [252]	-
	Induces autophagy-mediated cell death in liver cancer cells [253]	
Lys05	Inhibits cytoprotective autophagy in chronic myeloid leukemia [290]	-
	Inhibits cytoprotective autophagy in lung cancer cells [291]	
Mefloquine	Inhibits cytoprotective autophagy in colorectal cancer cells [292]	1
Mimulone	Induces autophagy-mediated cell death in lung cancer cells [293]	-
Neferin	Induces autophagy-mediated cell death in cervical cancer cells [240]	-
Nigericin	Induces autophagy-mediated cell death in glioblastoma cells [268]	-
Dactolisib (NVP-BEZ235)	Induces autophagy-mediated cell death in glioma [270]	22
Buparlisib (NVP-BKM120)	Induces autophagy-mediated cell death in breast cancer cells [281]	86
Pantoprazole	Inhibits cytoprotective autophagy in breast cancer cells, vulvar epidermoid carcinoma and prostate cancer cells [294]	16
Piplartine/piperlongumine	Induces autophagy-mediated cell death in osteosarcoma cells [248]	-
Quinacrine	Inhibits cytoprotective autophagy in glioblastoma cells [267]	4
Resveratrol	Induces autophagy-mediated cell death in breast cancer cells [9]	16
	Induces autophagy-mediated cell death in oral cancer cells [273]	
Rottlerin	Induces autophagy-mediated cell death in prostate cancer cells [295]	-
Siramesine	Inhibits cytoprotective autophagy in glioblastoma cells [266]	-
Sorafenib	Induces autophagy-mediated cell death in renal carcinoma cells [203]	836
SU11274	Induces autophagy-mediated cell death in lung cancer cells [215]	-
Suberoylanilide hydroxamic acid (Vorinostat/SAHA)	Induces autophagy-mediated cell death in glioblastoma cells [269]	256
Temozolomide	Induces autophagy-mediated cell death in glioblastoma cells [219]	953
	Induces cytoprotective autophagy in glioblastoma cells [223]	
Thioridazine	Induces autophagy-mediated cell death in glioblastoma cells [272]	1
Tioconazole	Inhibits cytoprotective autophagy in glioblastoma, colorectal and breast cancer cells [206]	-
Torin-1	Induces autophagy-mediated cell death in colorectal cancer cells [285]	-

* All studies at www.clinicaltrials.gov, on 6 August 2020. Trials identified by the search term “cancer” and the name of the drug.

## Abbreviations

3-MA: 3-methyladenine; 5-FU, 5-fluorouracil; AML, acute myeloid leukemia; AMPK, adenosine monophosphate-activated protein kinase; AraC, cytarabine; ATG, autophagy-related genes; ATO, arsenic trioxide; BAD, BCL-2 associated agonist of cell death; BAK, BCL-2 homologous antagonist/killer; BANCR, BRAF-activated lncRNA; BAX, BCL-2-associated X protein; BCL-2, B-cell lymphoma 2; BH-3, BCL-2 homology domain-3; BIF-1, bax-interacting factor-1; BNIP3, BCL-2 interacting protein 3; BRAF, B-Raf proto-oncogene; ccRCC, clear cell renal cell carcinoma; ChEH, cholesterol epoxide hydrolase; CMA, chaperone-mediated autophagy; c-MYC, cellular myelocytomatosis; CQ, chloroquine; CSCs, cancer stem cells; CXCR4, chemokine type 4 receptor; DAPK1, death-associated protein kinase 1; DDA, dendrogenin A; DEDD; death effector domain-containing; DRAM, damage-regulated autophagy modulator; EGFR, epidermal growth factor receptor; EMT, epithelial–mesenchymal transition; ER, endoplasmic reticulum; ERK, extracellular signal-regulated kinase; FDA, food and drug administration; GFP, green fluorescent protein; GTPase, guanosine triphosphatase; GX15-070, BH3-mimetic obatoclax; HCC, hepatocellular carcinoma; HCQ, hydroxychloroquine; HH, hedgehog; HOPS, homotypic fusion and protein sorting; HSC70, heat shock cognate 71 kDa protein; HSPA5, heat shock 70 kDa protein 5; HULC, highly upregulated in liver cancer; IGF-BP3, insulin growth factor binding protein 3; JNK, c-Jun N-terminal kinase; KRAS, Kirsten rat sarcoma; LAMP2A, lysosome-associated membrane protein type 2a; LC3, light chain 3; LDHA, lactate dehydrogenase A; LICs, leukemia-initiating cells; lncRNAs, long non-coding RNAs; LSCs, leukemic stem cells; LXR, liver-X-receptor; MAPK, mitogen-activated protein kinase; MEK, MAPK/ERK kinase; MDC, monodansylcadaverine; miRNAs, microRNAs; MST40, serine/threonine protein kinase; MTA1, metastasis-associated 1; mTOR, mammalian target of rapamycin; NAB, albumin-bound; NCCD, Nomenclature Committee on Cell Death; NOD, nonobese diabetic; NR4A1, nuclear receptor subfamily 4 group A member 1; NR4A3, nuclear receptor subfamily 4 group A member 3; SNAP29, synaptosome associated protein 29; NSCLC, non-small cell lung cancer; PAS, pre-autophagosomal structure; PDAC, pancreatic ductal adenocarcinoma; PE, phosphatidylethanolamine; P-gp, glycoprotein P; PI(3)P, phosphatidylinositol 3-phosphate; PI3K, phosphatidylinositol 3-kinase; PKR, protein kinase RNA-activated; PP2A, protein phosphatase 2A; PTEN, phosphatase and tensin homolog deleted on chromosome 10; RB1CC1, RB1 inducible coiled-coil 1; REDD1, regulated in development and DNA damage responses 1; ROS, reactive oxygen species; SAHA, suberoylanilide hydroxamic acid; SCID, severe combined immunodeficient; siRNA, small interfering RNA; Stx17, syntaxin17; TFEB, transcription factor EB; THC, Δ9 tetrahydrocannabinol; TIGAR, TP53-induced glycolysis and apoptosis regulator; TP53, tumor protein P53; TMZ, temozolomide; TXNDC17, thioredoxin domain-containing protein 17; ULK1, unc-51-like kinase; VAMP8, vesicle associated membrane protein 8.
